# Retraction: Plant and Fungal Diversity in Gut Microbiota as Revealed by Molecular and Culture Investigations

**DOI:** 10.1371/journal.pone.0346941

**Published:** 2026-04-13

**Authors:** The PLOS One Editors

The *PLOS One* Editors retract this article [1,2] due to concerns about compliance with the PLOS Human Subjects Research policy.

Specifically, the research ethics concerns included that the study involved a human participant but did not receive ethics approval from a Comité de Protection des Personnes, and that the ethics approval number N° 09–022, issued by the Ethics Committee of the Institute Fédératif de Recherche IFR 48 was also reported in at least 247 other studies despite apparent differences in the aims and objectives, study locations, study populations, age ranges, methodologies, types of samples collected, and types of consent described in these studies. S1 File contains a summary of articles citing ethics approval number N° 09–022 of which PLOS is aware.

A representative of the Aix-Marseille Université Ethics Committee stated that the institutional investigation into the ethics concerns concluded this article meets ethical standards. They commented that the study described in [1] is based solely on donated stool, that the stool sample described in this study is considered human waste, and that the study did not require ethics approval from a Comité de Protection des Personnes according to French law. The representative provided the ethics approval documents N° 09–022 for editorial review.

PLOS reviewed the documentation provided by the institution and concluded that the documents did not fully resolve the concerns. Specifically,

- Ethics approval document N° 09–022 issued on December 9, 2009 by the Comité d’Ethique de l’IRF48 for a study titled “*Culture diversifiée des bactéries dans les selles humaines*”. It allows for the use of clinical stool samples originating from the bacteriology laboratory of Timone Hospital, Marseille, France, as part of a study into the diverse culture of bacteria in human stools. The study described in the document does not appear to match the study reported in [1], which assesses plant and fungal diversity as opposed to bacterial diversity.
- In response to editorial queries neither the authors nor the institute clarified when the sample used in this study [1] was collected. This information is needed to evaluate the article’s compliance with the PLOS Human Subjects Research policy.
- PLOS identified potential competing interests between the committee that granted the ethics approval and one or more of the article’s authors.

In addition to the ethics approval concerns listed above, the findings of the study reported in this article [1] appear to be based on a stool sample obtained from a single individual, raising concerns about the reproducibility of the results reported in this study and the relevance of these findings to a larger population, and calling into question the article’s conclusions that the results reported in [1] constitute a baseline for further studies to assess eukaryotic diversity in healthy and diseased individuals from various geographical origins.

All authors either did not respond directly or could not be reached.

## Supporting information

S1 File Overview of 248 articles referencing ethics approval number N° 09-022. (XLSX)
